# Oxidative stress: role of physical exercise and antioxidant nutraceuticals in adulthood and aging

**DOI:** 10.18632/oncotarget.24729

**Published:** 2018-03-30

**Authors:** Carolina Simioni, Giorgio Zauli, Alberto M. Martelli, Marco Vitale, Gianni Sacchetti, Arianna Gonelli, Luca M. Neri

**Affiliations:** ^1^ Department of Morphology, Surgery and Experimental Medicine, University of Ferrara, Ferrara, Italy; ^2^ Department of Biomedical and Neuromotor Sciences, University of Bologna, Bologna, Italy; ^3^ Department of Medicine and Surgery, University of Parma, Parma, Italy; ^4^ CoreLab, Azienda Ospedaliero-Universitaria di Parma, Parma, Italy; ^5^ Department of Life Sciences and Biotechnology, Pharmaceutical Biology Laboratory, University of Ferrara, Ferrara, Italy

**Keywords:** exercise training, nutraceuticals, flavonoids intake, aging, antioxidant supplementation

## Abstract

Physical exercise is considered to be one of the beneficial factors of a proper lifestyle and is nowadays seen as an indispensable element for good health, able to lower the risk of disorders of the cardiovascular, endocrine and osteomuscular apparatus, immune system diseases and the onset of potential neoplasms. A moderate and programmed physical exercise has often been reported to be therapeutic both in the adulthood and in aging, since capable to promote fitness. Regular exercise alleviates the negative effects caused by free radicals and offers many health benefits, including reduced risk of all-cause mortality, sarcopenia in the skeletal muscle, chronic disease, and premature death in elderly people. However, physical performance is also known to induce oxidative stress, inflammation, and muscle fatigue. Many efforts have been carried out to identify micronutrients and natural compounds, also known as nutraceuticals, able to prevent or attenuate the exercise-induced oxidative stress and inflammation.

The aim of this review is to discuss the benefits deriving from a constant physical activity and by the intake of antioxidant compounds to protect the body from oxidative stress. The attention will be focused mainly on three natural antioxidants, which are quercetin, resveratrol and curcumin. Their properties and activity will be described, as well as their benefits on physical activity and on aging, which is expected to increase through the years and can get favorable benefits from a constant exercise activity.

## INTRODUCTION

Free radicals are generated during normal cellular function and are part of the natural physiological process of all living beings [1–3]. They normally act as both beneficial and toxic compounds, since they can be either helpful or harmful for the body [4]. When an overload of free radicals cannot gradually be processed or in case of a poor availability of the naturally occurring antioxidant body protection, free radicals accumulation in the body generates a phenomenon called “oxidative damage”, also known as “oxidative stress”, a term frequently used to imply random, indiscriminate damage to a wide range of biomolecules [5]. Oxidative stress is generally considered the starting point for the onset of several diseases and certainly plays a major role in the development of aging and chronic and degenerative disorders such as arthritis, autoimmune disorders, cardiovascular and neurodegenerative diseases, inflammation and cancer [6–14]. Reactive Oxygen Species (ROS) and Reactive Nitrogen Species (RNS) are the terms collectively describing free radicals and other non-radical reactive derivatives. Indeed, high levels of ROS can be lethal to cells. Modifications in cellular lipids are reported, as well as DNA and proteins degradation. ROS overproduction contribute to cell genomic instability and cancerogenesis, through the promotion of aberrant cell proliferation, apoptosis and uncontrolled cell growth [15].

Proteins are the biomolecules most frequently affected by oxidation and are believed to be the main target of ROS [16]. Elevated ROS levels can cause reversible post-translational modification of cysteine, selenocysteine, methionine and histidine. Furthermore, oxidative stress is responsible for epigenetic alterations, such as DNA hypomethylation, which also promotes genomic instability and protooncogene activation, as well as gene silencing due to regional hypermethylation of the promoter of tumor suppressor genes [17].

Oxidative stress and subsequent changes in signaling pathways could have different pathophysiological impacts at different stages of life [18] and interestingly, sex differences have been observed in oxidative stress generation [19]. Indeed, the brain is very sensitive to oxidative stress because of its high metabolic activity, high density of oxidizable substrates, and relatively low antioxidant defense [20]. Mitochondria are also particularly vulnerable to oxidative damage and are central to the theory of aging [20, 21]. With oxidative stress mitochondria membranes are seriously impaired, and in general a reduction of mitochondria biogenesis is reported [22]. Aging is characterized by a progressive decline in cellular function and body fitness together with increased risk of age-associated diseases, such as sarcopenia in skeletal muscle [23]. Therefore, in order to guarantee a good level of physical well-being, in the aging process it is also proposed to counteract the effects induced by oxidative stress with different strategies.

The aim of this review is to discuss the benefits deriving from a constant physical activity and by the intake of antioxidant compounds to protect the body from oxidative stress. Among a variety of antioxidants, the attention will be focused on three natural exogenous antioxidants, which are quercetin, resveratrol and curcumin. We will describe their properties and activity as well as their benefits on physical activity and on aging, which is expected to increase during the years and can get favorable benefits from a constant exercise activity. We focus on the most recent studies, predominantly performed in humans, reporting a definite, beneficial effect of the natural antioxidant intake associated with regular physical activity for adulthood and elderly subjects.

### Mechanisms of oxidation

Oxidation and reduction reactions, known as redox reactions, refer to all chemical reactions in which loss or gain of electrons occurs, thus modifying the oxidation number.

In a biological environment, oxidants, that accept electrons, and reductants, that donate electrons, are called pro-oxidants and antioxidants, respectively. A cell's redox state describes the pro-oxidant/antioxidant balance and plays an important role in the modulation of different signaling and metabolic processes [24]. During normal metabolism, oxygen is utilized in mitochondria for energy production. A small percentage of oxygen is not completely reduced, leading to the production of oxygen intermediates known as ROS [22]. At the same time, when reactants are derived from nitrogen, they are called reactive nitrogen species (RNS). Reactive species can be classified into two categories: free radicals and non-radical derivatives. The free radical group includes compounds such as the superoxide anion, nitric oxide or the nitric dioxide radicals. Most typical non radical reactive species relevant to biological systems are singlet oxygen, ozone (O_3_), hydrogen peroxide (H_2_O_2_), peroxynitrite (ONOO–), hypochlorous acid, organic peroxides and aldehydes. Cells and extracellular spaces are exposed to a large variety of reactive stimuli from both exogenous and endogenous sources. The exogenous sources include exposure to oxygen, radiation, air pollutants, drugs, alcohol, heavy metals, bacteria, viruses, sunlight, and food. Nonetheless, exposure to endogenous sources is much more important and extensive, because it is a continuous process during the lifespan. As mentioned before, the most vulnerable targets of reactive species are proteins, lipids and DNA [25]. ROS can oxidize proteins damaging their structure, impairing their functional activity and also affecting gene transcription [22]. Reactive species oxidize polyunsaturated free fatty acids and initiate lipoprotein oxidation, with consequent changes in fluidity and permeability of the cell membrane.

The best understood mechanism in which ROS, such as H_2_O_2_, achieve regulation of cellular function is through the redox-balance of cysteine residues within redox-sensitive proteins. Cysteines (Cys-SH) are readily oxidized by H_2_O_2_ to cysteine sulfenic acid (Cys-SOH) or cysteine disulphide (Cys-S-S-Cys). Exposure to ROS leads to oxidation of thiol groups of key cysteine residues in many proteins including kinases, phosphatases and transcription factors.

Several potential producers of ROS have also been identified in muscle cells. Among these are nicotinamide adenine dinucleotide phosphate (NADPH) oxidases (NOXs), phospholipase A2 (PLA2), xanthine oxidase (XO) and lipoxygenases. In addition to these intracellular sources, ROS has been shown to be produced from non-muscle sources. Strenuous exercise, a physical activity that requires lots of energy or force such as a marathon race, induces increased levels of pro-and anti-inflammatory cytokines, cytokine inhibitors, and chemokines. Thus, increased plasma levels of pro-inflammatory tumor necrosis factor (TNF)-α, interleukin (IL)-1β, IL-1 receptor antagonist (IL-1ra), TNF-receptors (TNF-R), IL-8 and macrophage inflammatory protein (MIP)-1 are found. Moreover, it has been reported that Interleukin-6 (IL-6), that acts both as a pro-inflammatory and anti-inflammatory multifunctional cytokine, is produced locally when contracting skeletal muscles in response to exercise and after strenuous exercise sessions [26–28]. The high concentration of IL-6 during intensive exercise is due to the fact that this interleukin acts also as a myokine, because it increases exponentially but proportionally to the duration of the exercise and the amount of muscle mass involved in the exercise. During exercise IL-6 is thought to act in a hormone-like manner to mobilize extracellular substrates and/or increase the supply of nutrients to the muscle [29]. Lipid peroxidation is the oxidative degradation of lipids [30–33], whose end products are reactive aldehydes, such as malondialdehyde (MDA) and 4-hydroxynonenal (HNE), the second one being known also as “second messenger of free radicals” and major bioactive marker of lipid peroxidation, due to its numerous biological activities resembling activities of reactive oxygen species [34, 35].

One of the major source of free radicals is immune system [36], and inflammation is the primary immune system reaction to eliminate pathogens or other stimuli in order to restore the cells to normal state or replace destroyed tissue with scar [37]. Indeed, when cells of an organ are damaged, the immune system cells become activated and trigger the production of free radicals to destroy damaged structures. However, the free radicals produced by the immune system against the damaged organ oxidize and damage neighboring healthy cells, generating inflammation.

(NADPH) oxidase is the main enzyme responsible for the production of ROS and RNS, such as H_2_O_2,_ hypochlorous acid (HOCl), ONOO–, hydroxyl (OH.) and ozone (O3). Inflammation reaction continues until the pathogens are removed and the tissue repair process be completed. The direct interaction between ROS and inflammation can lead to different disorders. Chronic inflammation predisposes cells for transformation due to induction of recurrent DNA damage by inflammatory cells, hence higher frequency of mutation [38]. In addition, chronic inflammation induces increase of growth factor production and growth-supporting stimuli. The presence of oxidative stress in myofascial tissues can also generate an inflammatory response [39]. Therefore, the immune status directly interplays with disease production processes [36]. The accumulation of oxidative stress, often also accompanied by dietary imbalances and incorrect lifestyles, can induce a high secretion of hormones with a pro-inflammatory action, such as Cortisol, Eicosanoids and Insulin [40].

Concerning signaling, nuclear factor (NF)-κB can be activated by ROS and it is known to play a critical role in mediating immune and inflammatory responses and apoptosis. NF-κB regulates the expression of a large number of genes, including several of those linked to diabetes complications [41]. In fact the aberrant regulation of NF-κB is associated with a number of chronic diseases, including diabetes and atherosclerosis. Recently, it was demonstrated that myostatin, that blocks muscle differentiation, is able to mediate ROS production via canonical Smad3, NF-κB and TNF-α in muscle cells [29]. In the absence of Smad3, myostatin induces ROS production through the activation of p38 and ERK mitogen-activated protein kinase (MAPK) pathways mediated via TNF-α, IL-6 and xanthine oxidase (XO). ROS generated by XO appears to be involved in the regulation of exercise-induced mitochondrial biogenesis via peroxisome proliferator-activated receptor-γ coactivator-1-α (PGC-1α). Free radicals can impair the DNA repair system and provoke mutagenesis, leading to decreased cellular and physiological functioning and promoting apoptosis and inflammation. The Ku heterodimer (Ku70/Ku80) is the main component of the non-homologous end-joining (NHEJ) pathway that repairs DNA double-strand breaks (DSBs) after oxidative stress. Ku binds the broken DNA ends and recruits other proteins to facilitate the processing and ligation of the broken ends [42, 43].

Among the other molecules with pivotal role in protection from oxidative stress, glutathione (GSH) plays a role as an intracellular protective substance in cells and serves as an effective oxygen radical scavenger. A decrease in cellular GSH content increases oxidative stress [44]. The endogenous thiols GSH and thioredoxin (TRX) systems play indeed a central role in the antioxidant defenses that control cellular events and regulate protection against oxidative stress, that alters normal redox control of cellular signaling, especially by disruption of thiol-redox circuits. In addition to their central role in supporting a large network of antioxidant defenses, GSH and TRX have a variety of biological functions such as regulation of enzymatic activity, receptors, transcription factors, and ultimately redox-sensitive signal transduction, short-term storage of cysteine, protein structure, cell growth, proliferation, and programmed cell death [45]. Superoxide dismutase (SOD) is another endogenous enzyme that alternately catalyzes the dismutation (or partitioning) of the superoxide (O_2_^−^) radical into either ordinary molecular oxygen (O_2_) or H_2_O_2_. Superoxide radical is produced as a by-product of oxygen metabolism and causes many types of cell damages, including an acceleration of age-related muscle mass loss, cancer and a reduced lifespan [46]. Finally, catalase is an endogenous enzymatic antioxidant that converts hydrogen peroxide into water and oxygen gas. Catalase possesses one of the highest turnover numbers of all enzymes. Indeed, one catalase molecule can convert millions of hydrogen peroxide molecules to water and oxygen each second [47, 48].

### Physical exercise as an antioxidant

Physical activity is defined as any bodily movement produced by skeletal muscles that results in energy consume, which may be unstructured, it can be an everyday life activity, an exercise that includes prearranged, deliberate and repetitive activity, grassroots and competitive sports and a regular physical activity of moderate intensity such as walking, cycling or sports that brings significant health benefits [49]. The term “physical activity” should not be confused with the term “exercise”, which is a sub-category of physical activity and is characterized by being planned, structured, constant and aimed at improving or maintaining one or more aspects of physical fitness. Both moderate and vigorous physical activity bring health benefits. Physical efforts and skills can be involved in the common term of “Sport”, a human activity capable of achieving a result requiring physical exertion and/or physical skill, which, by its nature and organization, may be competitive and is generally accepted as being a sport.

In order to counteract the negative effects and toxicity of oxidative stress on health, subjects at any age, with particular attention to aging, can benefit from constant, therefore repeated over time, physical activity that can alleviate the harmful effects caused by free radicals. However, although reactive species are associated with harmful biological events, they are essential in cellular development and optimal function. Indeed, cells have evolved strategies to utilize reactive species as biological stimuli. They act as subcellular messengers in important molecular signaling processes and modulate enzyme and gene activation. ROS are involved in the immune response of cells and drug detoxification, they are a requisite for vasodilation, optimal muscular contraction and initiation of apoptosis [22]. Moreover, accumulating evidence suggests that ROS are generated during exercise and modulate the level of muscle contraction. Modest ROS supplementation causes increase to force. However, relevant rise in ROS production that occurs during strenuous exercise contributes to the development of acute muscle fatigue [50].

Physical activity improves antioxidant defenses and lowers lipid peroxidation levels both in adult and in aged individuals [51]. Elderly physically active individuals show antioxidant activity and lipid peroxidation levels similar to young sedentary subjects, emphasizing the importance of regular physical activity to decelerate the aging-associated impairment process.

Moderate exercise and an active lifestyle have been demonstrated to be useful not only in the prevention of oxidative stress, but also in the primary and secondary protection from cardiovascular disorders, type II diabetes, metabolic syndrome and neurodegenerative diseases like Alzheimer's disease [52]. The beneficial effects of exercise are also reflected in the release of myokines. These molecules exert auto-, para- and/or endocrine effects and include cytokines, interleukins such as IL-6 and other peptides that are produced, expressed, and released by muscle fibers and have a role in the protection against diseases associated with low-grade inflammation such as atherosclerosis [53, 54].

The extent to which reactive species are or helpful or harmful depends on the exercise duration, intensity, fitness condition and nutritional status of the individual [55].

The antioxidant defense tools of the body consist of antioxidant enzymes (superoxide dismutase, catalase and glutathione peroxidase, etc.) and non-enzymatic antioxidants (Coenzyme Q10, glutathione, uric acid, lipoic acid, bilirubin, etc.). The exercise-induced ROS generation results in increased activity of enzymatic antioxidants, which then lead to an increased resistance to oxidative challenges, including a wide variety of oxidative stress-related diseases, including cardiovascular diseases, acquired neurodegenerative disorders (Alzheimer's and Parkinson's disease), asthma, diabetes and mitochondrial myopathies. CoQ10, also known as ubiquinone, is a fat-soluble molecule present in most eukaryotic cells, primarily in mitochondria. It is a component of the electron transport chain and plays a part in the cellular energy production. Its reduced form, ubiquinol, acts as an important antioxidant in the body. CoQ10 is synthesized endogenously, and its dietary uptake is limited.

It has been reported that strenuous exercise, reported as at least thirty minutes of intense and close to the limit muscle contractile activity, increases oxidant production in muscle, limiting performance [56]. Chronic exposure to high levels of ROS can become toxic, exhausting the enzymatic and non-enzymatic antioxidant system and leading to impaired cellular function, macromolecule damage, apoptosis, and necrosis. Therefore, excessive physical exercise is detrimental to untrained individuals, but progressive training allows the cells to more easily detoxify a larger amount of ROS. An excessive physical activity could be detrimental when it induces an altered hormonal activity, changes of the sleep-wake rhythm, of the appetite, alterations of the arterial pressure and of the heart rate. Indeed, those who are over-trained often find an increase in their heartbeats even at rest, struggle to fall asleep, are nervous and/or depressed, can be hypotensive and have a less efficient immune system.

Thus, since it has been reported that subjects involved in regular exercise, due to an adaptive response, demonstrate higher levels of mitochondrial content and accumulate lower levels of ROS at the given intensity than those who are untrained, the rationale is that both younger and elder population can take advantage of a constant physical activity in order to favor a more rapid recovery of the oxidation generated by strenuous exercise bouts, often referred to a maximal aerobic test, and thus to protect the body from oxidative damage.

### The exogenous antioxidants

**Figure 1:**
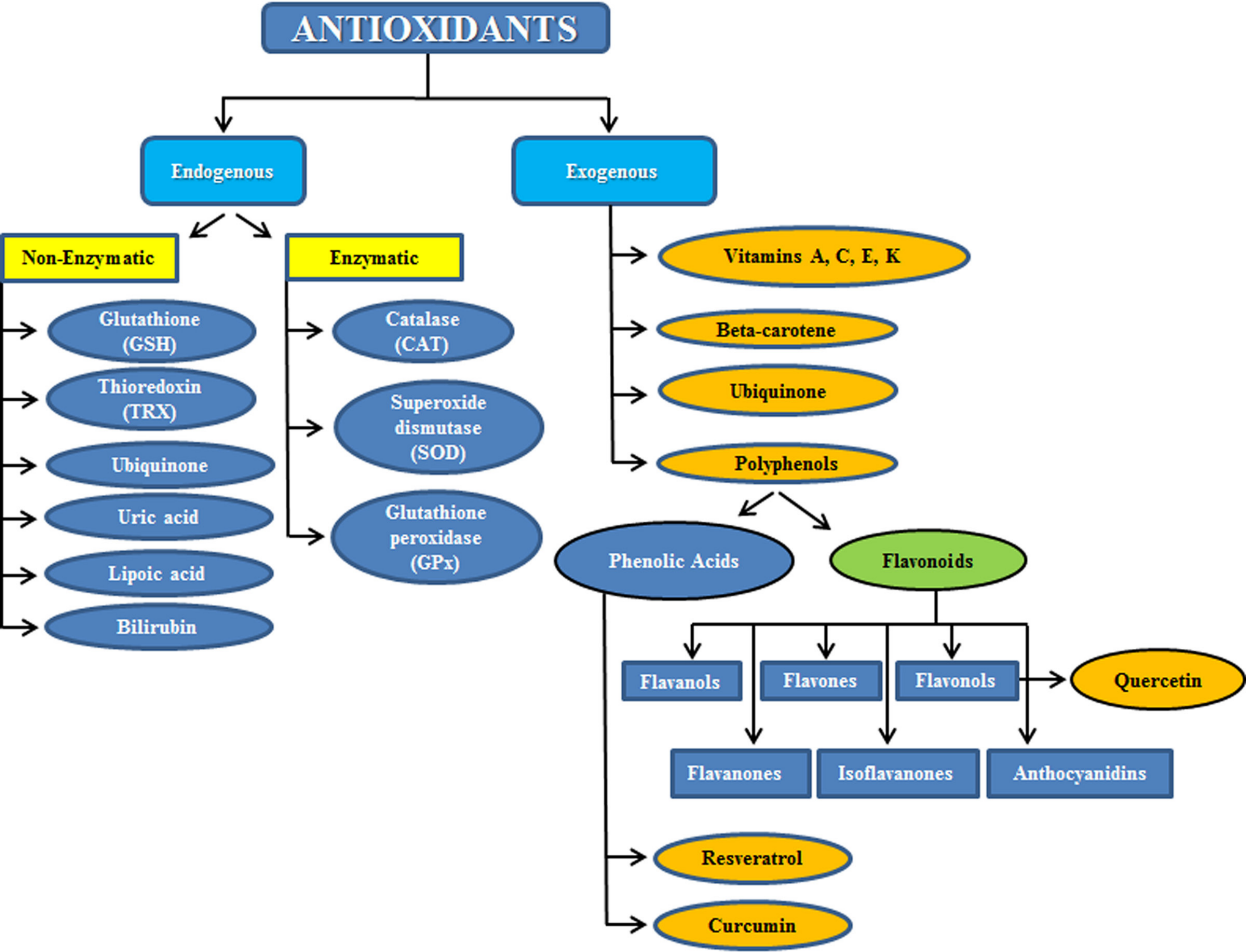
Subdivision between endogenous and exogenous antioxidants

Besides the endogenous enzymatic and non-enzymatic antioxidant defenses that the human body develops to cope with the excess of free radicals produced upon oxidative stress, and besides the protective mechanisms of scavenging or detoxifying ROS, blocking ROS production or sequestering transition metals, the body exploits also other antioxidants which are normally supplied within the diet and which are called exogenous antioxidants (Figure 1).

Nutritional antioxidants act in different mechanisms and compartments, but are mainly free radical scavengers: 1) they neutralize free radicals, 2) they repair oxidized membranes, 3) they decrease reactive oxygen species production, 4) via lipid metabolism, short-chain free fatty acids and cholesteryl esters neutralize reactive oxygen species [57].

Exogenous antioxidants have generated growing interest in preventing or reducing oxidative stress, in decreasing muscle soreness and physical stress, and in ameliorating sport performance [7]. The exogenous antioxidants act in addition to the endogenous ones, and the most known are tocopherols (vitamin E), ascorbic acid (vitamin C), carotenoids (β-carotene), ubiquinone and polyphenols [58, 59].

Vitamin E refers to a group of fat-soluble compounds that include tocopherols and tocotrienols. α-Tocopherol is the most biologically active form, has been shown to protect the cells from lipid peroxidation [60] and has been shown to have a role in the prevention of chronic diseases associated with oxidative stress.

Vitamin C or L-ascorbic acid is a co-factor in a range of essential metabolic reactions in humans (e.g. collagen synthesis). This water-soluble vitamin is produced endogenously by almost all organisms, some species of birds and several mammals, excluding humans. L-ascorbate, an ion form of ascorbic acid, is a strong reducing agent and its oxidized form is reduced back by enzymes and glutathione [22]. β-Carotene belongs to a group of red, orange and yellow pigments called carotenoids [61]. Others include α-carotene, β-cryptoxanthin, lycopene, lutein and zeaxanthin. These fat-soluble substances are found in plants and play a role in photosynthesis. β-Carotene is the most active carotenoid; after consumption it is converted to retinol, a readily usable form of vitamin A. β-carotene possesses antioxidant properties, anticancerogenic effects and has positive effects on the immune system. CoQ10 is synthesized endogenously, but it can be taken also as exogenous antioxidant [62, 63] and is well tolerated over a wide dosage range, with minimal side effects [64]. Some authors reported that CoQ10 may have a beneficial effect in the treatment of oxidative phosphorylation disorders [65] and can reduce oxidative stress in glaucoma patients [66].

Polyphenols are a group of water-soluble, plant-derived substances, characterized by the presence of more than one phenolic group [67]. Polyphenols are divided into two sub-categories: flavonoids and phenolic acids. Although there may be some evidence to support acute antioxidant supplementation immediately before certain types of intense exercise, where performance is fundamental, it is a much more common practice for athletes to continuously take antioxidants throughout the training period.

Among antioxidants, flavonoids, a polyphenol class of pigments generally ubiquitous in the Plant Kingdom usually occurring in flowers, fruits and seeds, has been demonstrated to provide many health benefits and to influence exercise performance for athletes and for subjects non necessarily in constant training, such as elderly people [68]. Flavonoids include flavonols (quercetin), flavones (luteolin), flavanones (naringenin), anthocyanidins (cyanidin) and isoflavones (genistein). The other classes of non-flavonoid compounds involve low-molecular weight phenolic acids, stilbenes, chalcones, lignans and coumarins [69].

In this review we will describe the flavonoid quercetin and the two non-flavonoid polyphenols resveratrol and curcumin. The choice of these three antioxidants is given by the fact that they have excellent antioxidant and anti-inflammatory properties in both *in vitro* and *in vivo* models, are natural substances with promising therapeutic activities and potential health benefits, are easily recoverable from plants, food and other supplements, enter easily in a common daily diet and up to date display no toxicity for the body. We will examine their properties and activity as well as their benefits on sport performance and on aging, which is often associated with decreased vascular function partially due to oxidative stress, muscular weakness, fatigue and cognitive decline and which is expected to increase through the years and can get favorable benefits from a constant exercise activity.

### Quercetin

Quercetin (3,3′,4′,5,7-pentahydroxylflavone) is a natural bioactive flavonoid found in a wide variety of cultivated plants and derived foods, such as nuts, grapes, onions, broccoli, apples and black tea [70]. Its amounts in vegetables was found to be below 10 mg/kg, but it reaches up to 40 mg/kg in beans and apples and up to 100 mg/kg in onions.

The optimal absorption of quercetin glucoside, a quercetin derivative, ranges from 3% to 17% in healthy individuals receiving a dose of 100 mg. Recent data indicate that the bioavailability of quercetin increases with the co-ingestion of fatty acids [71].

The average half-life of quercetin is a few hours, and the amount recovery of this flavonoid in plasma, urine, feces and exhaled air is very variable, depending on the subjects and on its stability at different temperatures [72].

This flavonoid is known to exert a valuable antioxidant activity [73]. Antioxidant properties of quercetin are attributed to its chemical structure, particularly to the presence and position of the hydroxyl (-OH) groups, responsible of the protection against free radical injury through a radical scavenging mechanism [74]. It has been reported that quercetin is able to regulate the transcription factor AP-1 [75], involved in the expression of genes associated with cell growth and cellular stress. Recent discoveries have shown that quercetin induces and activates Sirtuin-1 (SIRT1), correlated to skeletal muscle function and mitochondrial formation [76].

Isoquercetin (glycosylated quercetin) is more completely absorbed than the quercetin aglycone form, most probably because the carbohydrate(s) linked to the flavonoid (glycone) exert an important vehiculation role in the absorption due to the more hydrophilicity of the glicoside. Human subjects can absorb significant amounts of quercetin from food or supplements, with a reported half-life ranging from 11 to 28 h [77, 78].

Estimated daily quercetin intakes have been observed in the range of 3–40 mg (expressed as aglycone equivalents) in Western diets [79]. On the other hand, it has been estimated that quercetin intake of consumers of fruits and vegetables is about 250 mg per day. In dietary supplements, recommended daily doses of quercetin are usually in the range of 500–1000 mg. In a recent review dealing with the use of quercetin in prostatic diseases (quercetin dose: 1000–1500 mg) it was mentioned that side effects with quercetin therapy were rare. Some patients experienced nausea if the substance was taken on an empty stomach, but no particular adversal effects were detected [79]. This highlights the fact that high quantities of this flavonoid is safe, with no relevant side effects.

*In vitro* and *in vivo* animal model studies indicate that quercetin has not only antioxidant effects but other multiple biological effects such as anti-inflammatory, anticarcinogenic, antiviral, psychostimulant, cardioprotective, neuroprotective, antipathogenic and immune regulatory [80–86]. Moreover, a recent study published the cancer preventive effects of this flavonoid in smokers affected by pancreatic cancer [87]. Some evidence for the potential of this flavonoid to decrease pancreatic cancer risk is also available from animal studies and *in vitro* systems. In an animal study, quercetin decreased primary pancreatic tumor growth, increased survival, and prevented metastasis [88].

### Resveratrol

Resveratrol is a polyphenol chemically characterized as 3,5,4′-trihydroxystilbene occurring in the seeds and skins of grapes, red wine, mulberries, blueberries, cranberries, peanuts and, in particular, in the roots of the cultivated knotweed *(Polygonum cuspidatum)* [89]. Red wine and knotweed are the most common natural source of resveratrol. Many *in vivo* and *in vitro* studies reported different important properties of this natural compound. Resveratrol is non-toxic, easily absorbed and well tolerated by humans. The metabolism of resveratrol is high, leading to the production of conjugated sulfates and glucuronides, which retain some biological activity and accumulate in intestinal cells and in the liver [90].

Concerning its antioxidant activities, *in vitro* evidence shows that resveratrol can scavenge hydroxyl radicals and prevent oxidative DNA damage [91] and it induces the upregulation of SOD 1, 2 and 3, catalase and glutathione peroxidase (GPX) protecting the organism from oxidative damage. Resveratrol displays antioxidant activity in plasma and serum samples of patients and animal models affected by lung/heart tissue of pulmonary hypertension (PH) [92]. Moreover, it was recently demonstrated that 8 weeks of supplementation with 800 mg/day resveratrol has an antioxidant effect in the blood and PBMCs of patients affected by type II diabetes (T2D), a pathogenesis in which oxidative stress has a pivotal role. The well tolerated resveratrol increased plasma total antioxidant capacity and total thiol content. Furthermore, the expression of Nrf2 and SOD was significantly increased after resveratrol consumption [93]. It has also been reported that NADPH oxidase, an enzyme responsible for ROS production in the vasculature, is suppressed by this polyphenol [94].

Similarly to other polyphenols, besides the antioxidant property, resveratrol is known to have neuroprotective, cytoprotective, antithrombotic, DNA protective, antiinflammatory and anticancer effects [95–98]. Interestingly, resveratrol has also an anti-aging, longevity and cell reprogramming effects, documented mainly in murine models [96].

### Curcumin

Curcumin, also chemically known as diferuloylmethane, is a bright yellow polyphenol found in the rhizome of *Curcuma longa* (turmeric) and it is typically used in the Middle East and Asian countries [99]. This polyphenol has been shown to target multiple signaling molecules. Among them, Phosphorylase Kinase (PhK) involved in promoting photocarcinogenesis through activation of NF-κB -dependent signaling pathways [100] and IFN-γ signaling [101].

To improve curcumin absorption, pharmacological formulations have been designed with nanoparticles and liposomes. As an alternative, other food can be mixed with this spice to increase its absorption: cyclodextrin (CD) which is usually derived from starch, and piperine, that is usually mixed with curry. Actually, liposomal encapsulation is considered one of the most effective drug carrier, due to its ability to solubilize hydrophobic compounds and to pharmacokinetic properties [102]. Most of curcumin is metabolized in liver and intestine, but a small amount remains detectable in the other organs [103].

Curcumin is well known to be a natural compound exerting antioxidant effects [104, 105]. Due to its particular chemical structure, curcumin is indeed a scavenger of reactive oxygen and nitrogen species [106]. In addition, curcumin is a lipophilic compound, which makes it an efficient collector of peroxyl radicals. Curcumin can modulate the activity of GSH, catalase, and SOD enzymes active in the neutralization of free radicals and it can inhibit ROS-generating enzymes such as lipoxygenase/cyclooxygenase and xanthine hydrogenase/oxidase [99].

It was also shown that curcumin can benefit inflammatory conditions by blocking NF-κB activation, mediates anticancer effects by modulating multiple cell signaling pathways, such as PI3K/Akt/mTOR pathway, and has neuroprotective effects by preventing aggregation of senile plaques (β-amyloid, Aβ) *in vitro* and in cell cultures [69, 104, 107].

### Quercetin, resveratrol and curcumin and their synergism with physical exercise

The role of nutraceutical formulations in improving exercise performance arouses increasing interest for researchers. Literature suggests that polyphenol nutraceuticals and their subclass flavonoids have a protective role against exercise-induced muscle injury, just due to their antioxidant and anti-inflammatory properties [108]. One rationale of the nutraceutical protective role against exercise-induced muscle injury and muscle cell damage is given by the fact that quercetin, resveratrol and curcumin activate SIRT1 [109, 110]. SIRTs are a family of NAD^+^ dependent histone/protein deacetylases highly conserved through bacteria and eukaryotes [111]. SIRT1 has been demonstrated to inhibit oxidative stress and inflammation [112, 113] and deacetylates a wide range of substrates, including p53, NF-κB, FOXO transcription factors, Ku-70 and PGC-1 α, with roles in cellular processes ranging from energy metabolism to cell survival. Many studies in recent years have highlighted the correlation of SIRT1 activity with AMPK and mTOR pathway [114–116]. These two kinases play a key role in many pathophysiological processes, such as mitochondrial biogenesis, and their correct activation (for AMPK) or inhibition (for mTOR) is fundamental in such processes. Many works have demonstrated that polyphenols are able to modulate SIRT1 and that this activity is related to the increase of AMPK and the decrease of mTOR signaling [117]. In an *in vitro* experiment, Giovannini et al. [111] tested the effects of different concentrations of resveratrol, quercetin and curcumin in a human cervical carcinoma cell model. Results reported that, after treatments of 3, 6 and 24 h, the expression of SIRT1 was significantly increased in all experimental groups compared with the control group, demonstrating the ability of these compounds to modulate SIRT1. The individual administration of quercetin resulted in a statistically significant increase in AMPK activation and mTOR inhibition, whereas their associated administration did not reveal a synergistic effect. Therefore, SIRT1 may have a preventive or pro-curative pharmacologic application in many disorders in which SIRT1 has a key role, such as cancer, age-related or metabolic disorders. SIRT1 has different properties, such as the regulation of energy metabolism, cell survival, modulation of several transcription factors, and acts also as a metabolic sensor and a regulator of survival under stress conditions, such as caloric restriction and oxidative stress. The fact that resveratrol, quercetin and curcumin are able to stimulate SIRT1 confirms the stimulating action of these nutraceuticals on SIRT1.

Concerning the flavonoid quercetin, to evaluate its supplementation effect on endurance performance and antioxidant status, young male runners received for six weeks quercetin (1000 mg/day) or placebo while maintaining their current training schedules. At the end of the supplementation period, results indicated that there was a significant relationship between quercetin supplementation and the statistically significant decreasing trend in MDA levels. Quercetin supplementation during training for 6 weeks can therefore decrease oxidative stress in well-trained long distance runners, implying the potential for improved endurance performance [118].

The exercise-induced lipid peroxidation was also reduced by the combination of quercetin and resveratrol in fourteen athletes randomly assigned to take these two compounds the week before exercise. Blood was taken at baseline, pre-exercise, immediately after exercise, and 1 h after exercise. Plasma was analyzed for oxidative stress (F2-isoprostanes and protein carbonyls), antioxidant capacity (ferric-reducing ability of plasma (FRAP)), Trolox equivalent antioxidant capacity (TEAC), oxygen radical absorptive capacity (ORAC), inflammation (cytokine interleukin (IL-8)) and C-reactive protein (CRP). Data analysis indicated that the supplementation of quercetin and resveratrol significantly reduced exercise-induced lipid peroxidation [119].

In another study, conducted among 60 healthy subjects non-professional athletes with regular exercise, it was seen that eight-week supplementation with quercetin and vitamin C was effective in reducing oxidative stress and reducing inflammatory biomarkers including IL-6 [120].

Quercetin positively regulates the expression of genes associated with mitochondrial biogenesis and skeletal muscle function (PGC-1α and SIRT1 mRNA) [80]. PGC-1α has been reported to play an important role in stimulating mitochondrial biogenesis following physiological demands and nutritional inputs, such as exercise and the dietary flavonoid resveratrol [121].

Resveratrol has been shown to enhance training-induced changes in cardiovascular function, exercise performance and retardation of atherosclerosis [122]. Resveratrol administration seems to induce a higher aerobic capacity in mice, as shown by the increased running time and oxygen consumption in muscle fibers [123]. Hart et al. suggested that resveratrol supplementation enhanced the effects of exercise on endurance capacity, and this was shown in rats which already had a high level of aerobic endurance [124]. These findings suggest that resveratrol could be used as a performance enhancer [125, 126]. Supplementing rats with resveratrol could deeply improve exercise performance, muscle strength and regeneration, increasing skeletal muscle mitochondrial biogenesis and fatty acid oxidation in many tissues [127]. Increasing doses of resveratrol can strengthen the heart contraction force and prepare it to adapt to more intense efforts and can improve cardiac and skeletal muscle energy metabolism inducing vasorelaxing effects [128].

Curcumin has been demonstrated to have a relevant role in muscle regenerating and in treating muscle injuries. In a recent work, male Wistar rats were treated for a prolonged period with curcumin and subjected to an exercise protocol for 6 weeks on a motor-driven rodent treadmill. After the last week animals were sacrificed and serum glucose, lipid profile, aspartate transaminase, alanine transaminase, urea, and creatinine levels were measured. The oxidative stress marker MDA level in muscle tissue was measured by high-performance liquid chromatography. Results reported that the combination of exercise with curcumin accelerated mitochondrial biogenesis in the skeletal muscle, regulated the NF-κB and PGC-1α pathways, reduced the MDA level and increased cytosol and the NAD+/NADH ratio and SIRT1 protein in muscle, thus highlighting the antioxidant ability of curcumin in improving physical performance [129].

In a small group of young men exposed to eccentric exercise, the prolonged curcumin ingestion attenuates some aspects of muscle damage markers such as maximal voluntary contraction strength (MVC) and creatine kinase (CK) activity expression, at different times of eccentric exercise. MVC is expressed as the maximum force produced by a human subject in a specific isometric exercise. Curcumin intake has some beneficial effects on recovery of eccentric exercise-induced muscle damage [130].

Taken together, the intake of quercetin, resveratrol and curcumin appears to provide a significant benefit on physical exercise, in muscle regenerating, in treating muscle injuries and in potentiating the mitochondrial biogenesis, in which the antioxidant mechanisms have a pivotal role. Therefore, exogenous antioxidant supplementation could help athletes with initially low antioxidant levels to improve their antioxidant status.

### Quercetin, resveratrol and curcumin and aging

Natural products or nutraceuticals have been shown to have anti-aging activity, and their consumption is highly recommended as a preventive antioxidant tool, together with a constant and adequate physical activity [131]. In the aging process, differently than in the younger population, accumulation of mitochondria DNA mutations, impairment of oxidative phosphorylation and an imbalance in the expression of antioxidant enzymes result in further overproduction of ROS. The excess of ROS such as superoxide and hydrogen peroxide, that are often released by aging vasculature, compromise the vasodilatory activity of NO and facilitate the formation of peroxynitrite, inducing oxidative stress. Moreover, since it has been proposed that NO inhibits endothelial senescence [76], the interference of ROS on the NO signaling system would influence endothelial aging [132].

During aging, regular exercise is also important because it offers many health benefits, including reduced risk of all-cause mortality, chronic diseases, and premature death [133].

Moreover, physical exercise leads to an increase in antioxidant protections of the organism in younger as well as in older subjects [134, 135]. An acute level of exercise increases antioxidant activities in skeletal muscle, heart, and liver [136] thus limiting free radical production and oxidative damage. This has also been demonstrated in older people who benefit from exercise inducing a strengthening in the antioxidant defense system [137].

Oxidative stress has been reported also for neuronal cell injury, which typically occurs in aging [138]. Aging and brain aging are associated with free radicals, and an altered balance level has been reported particularly to Alzheimer's disease, a chronic neurodegenerative disorder that leads to a progressive synapse loss, memory decline, speech and personality changes [68].

Since different studies obtained from animal models demonstrated that a constant supplementation with antioxidants can prevent the oxidative stress in the central nervous system and more in general in the whole body, the rationale is that elderly people can benefit from the intake of different exogenous antioxidants that might ameliorate neural decay caused by oxidative damage, that generally induces a cellular aging process [139].

Quercetin reduced lipid peroxidation products and increased levels of the antioxidant glutathione in the brains of mice [140].

Quercetin has protective effects mainly for the treatment of neurodegenerative disorders and cerebrovascular diseases, both *in vitro* and *in vivo* [141]. This flavonoid, as well as Isoquercetin at different concentrations is neuroprotective for Alzheimer's patients and could act as promising memory enhancers and cholinomimetics [142]. Quercetin is able to stimulate neurogenesis and neuronal longevity by modulating a broad number of kinase signaling cascades such as phophoinositide 3- kinase (P13-kinase) and Akt and PKC [141]. Quercetin has also been well reported for its ability to reverse cognitive impairment and memory enhancement during aging [143].

As for quercetin, also resveratrol has neuroprotective properties by reducing oxidative damage and chronic inflammation, by improving vascular function and by activating longevity genes and SIRTs [144]. SIRTs are necessary for DNA repair mechanisms, inflammation control and antioxidative defense. In aged mice, Murase et al. [145] showed that a combination of resveratrol intake and habitual exercise suppressed aging-associated decline. At 18 weeks of age, senescence-accelerated prone mice (SAMP1) were fed with resveratrol and started using treadmill running according to a 5 days running program. It emerged that resveratrol effectively suppressed the aging-associated decline in endurance capacity. In addition, mice had significantly attenuated senescence-related declines in whole body oxygen consumption and fat oxidation, and hemoglobin levels were increased, which might lead to an improvement in aerobic exercise capacity. Moreover, Ryan et al. [146], in the exercised muscles of aged mice compared with non-exercised mice, demonstrated also that 10 days of resveratrol supplementation diminished the basal levels of oxidative stress associated with aging. Resveratrol supplementation prevented the exercise-induced decrease in the reduced glutathione/oxidized glutathione (GSH/GSSG) ratio in the gastrocnemius muscle, attenuated the increase in xanthine oxidase, hypoxanthine, and xanthine activity and significantly reduced H_2_O_2_ by 15% [146, 147].

Resveratrol is involved in suppression of drug-induced cardiotoxicity, especially in rat cells [148]. It induces SIRT1 expression which is also important in cardiac cells surviving and, in mice, suppresses the induction of cardiotoxicity induced by doxorubicin [149].

Elderly people are also affected by muscle fatigue [150]. A recent study documented the effect of 12 weeks exercise on a group of men and women in the range of 65–80 years. The aim of the study was to examine if, during exercise, the concomitant supplementation of resveratrol increased mitochondrial density, with a further decrease of muscle fatigue resistance. Data showed that resveratrol could effectively reverse sarcopenia than exercise alone, and this was demonstrated by muscle greater fiber sizes and muscle power [151].

Resveratrol can be implied in anti-aging actions by influencing the mitochondrial environment and metabolic diseases, by regulating the levels of some inflammatory mediators and cytokines and by modulating lipolysis [125, 152, 153]. Mitochondrial dysfunction has been proved to be associated with aging and disease development [154], and it was seen that transient increase in the mechanism of autophagy represents a compensatory response to this dysfunction [155]. Multiple works indicate that Autophagy-Related Genes (ATG) or other proteins required for autophagy induction, such as SIRT1, display reduced expression in aged tissues and therefore autophagy diminishes with aging [156]. This applies, for instance, to normal human brain aging (in which the autophagy markers Atg5, Atg7, and Beclin1 are downregulated) to insulin resistance and metabolic syndrome (in which Sirtuin1 is downregulated) or to osteoarthritis, where a downregulation of ULK1, Beclin1, and LC3 occurs [156, 157]. Resveratrol can act as an autophagy enhancer [158–162], especially in the treatment of Alzheimer's Disease, characterized by an increment of Inositol trisphosphate (IP_3_) receptor signaling [158]. The autophagy-mediated neuroprotective effects by resveratrol are given by the fact that this polyphenol is able to activate SIRT1 and deacetylate histone acetylases through AMPK/SIRT1 signaling pathway. Moreover, resveratrol can directly activate autophagy and exert neuroprotective effect by inhibiting also the mTOR signal pathway. In spine disc nucleus pulposus (NP) tissue samples obtained from patients who underwent discectomies, resveratrol could induce autophagy and delay the progression of disc dysfunction, protecting from mitochondrial dysfunction and from cell apoptosis under oxidative stress [162].

Furthermore, resveratrol maintains the vascular fitness through its antioxidant and anticoagulant activities, and on the other hand is relevant in blocking the formation of new blood vessels, in inhibiting the VEGF release and attenuating Hypoxia-Inducible Factor (HIF-1α) in different tumor cells [163].

It is reported that also curcumin possesses anti-aging properties, although few data are still available [131]. As already mentioned before, one of the typical feature of aging is sarcopenia, which can impair the ability to perform routine activities, with a negative impact on quality of life [164]. Curcumin is important for older people in preventing muscle cracking and in inhibiting NF-κB levels which is normally responsible for the onset of sarcopenia. Curcumin increases anti-oxidant protection by improving strength and physical performance in elderly subjects, potentially preventing the onset of sarcopenia and blocking the progression of proinflammatory signaling [164].

Its antioxidant properties have been also studied in erythrocytes isolated from Wistar rats, where the plasma membrane redox system (PMRS) was analyzed. PMRS is an electron transport chain system ubiquitously present throughout all cell types and an altered form of PMRS activity and redox status are associated with the pathophysiology of several health complications including diabetes. PMRS plays an important role in regulating antioxidant status of the plasma during aging and progression of age associated diseases. Effects of curcumin were also evaluated on level of glutathione (GSH) and the plasma oxidant potential measured in terms of plasma ferric equivalent oxidative potentials (PFEOP). Results showed that curcumin significantly modulated the PMRS activity in a dose-dependent manner, interacting with amino acids at the active site cavity of cytochrome b 5 reductase, a key constituent of PMRS. Curcumin also increased the GSH level in erythrocytes and plasma while simultaneously decreasing the pro-oxidant potential (PFEOP) of plasma, explaining partially the role of curcumin health beneficial effects [165].

In a recent work it was seen that in healthy middle-aged and older adults, who were sedentary or moderately physically active, 12 weeks of curcumin supplementation improves artery endothelial function by increasing vascular nitric oxide bioavailability and reducing oxidative stress [166]. Moreover, it was seen that an active metabolite of curcumin, Tetrahydrocurcumin (THC), possesses extremely strong antioxidant activity compared to other curcuminoids. The antioxidant role of THC has been implicated in recovery from renal injury in mice and in anti-inflammatory responses [167].

Oxidative stress can be involved in age-related cerebrovascular dysfunction, which contributes to stroke, cerebral amyloid angiopathy, cognitive decline and neurodegenerative diseases, up-regulation of mitochondrial uncoupling protein 2 (UCP2) plays a crucial role in regulating reactive oxygen species (ROS) production. Dietary patterns are widely recognized as contributors to cardiovascular and cerebrovascular disease. Pu et al. [168] tested the hypothesis that dietary curcumin, in 24-month-old male rodents, could ameliorate aging-related cerebrovascular dysfunction via UCP2 up-regulation. It was seen that curcumin administration for one month remarkably restored the impaired cerebrovascular endothelium-dependent vasorelaxation in aging rats and up-regulated UCP2 and reduced ROS production, thus representing a promising lifestyle intervention for preventing age-related cerebrovascular disturbances. The description of the effects of Quercetin, Resveratrol and Curcumin observed in adulthood, elderly people and animal models and described in this review are reported in Table 1.

**Table 1:** Description of the effects of quercetin, resveratrol and curcumin observed in adulthood, elderly people and animal models, the antioxidant dose administration, the proposed physical exercise intervention programs or experiments and the obtained results

Quercetin, Resveratrol and Curcumin in Adulthood			
Antioxidant	Antioxidant Supplementation	Physical Activity Intervention	Outcome
Quercetin	- 6 weeks of Quercetin supplementation (1000 mg/day)	1) 10 km run and aerobic exercise program of at least 90–180 minutes per week REF [118]	1) Decreased oxidative stress
	- 225 mg Quercetin for 6 days and 450 mg on day 7 just prior to exercise	2) 1 h run Program in fourteen subjects REF [119]	2) Reduced exercise-induced lipid peroxidation
	- 8 weeks 500 mg quercetin and 250 mg vitamin C as pro-oxidant (Q+C), 500 mg of quercetin alone (Q), 250 mg of vitamin C alone (C)	3) Non-professional athletes with regular exercise REF [120]	3) Reduced oxidative stress and inflammatory biomarkers
Resveratrol	- 9 weeks 100 mg of resveratrol/kg of body mass/day.	1) 9-week period on running wheels REF [123]	1) Increment of aerobic capacity
	- 12 weeks 146 mg of resveratrol	2) 12 weeks treadmill running REF [124]	2) Enhanced exercise performance
Curcumin	- 20 mg of curcuminoids daily for 6 weeks	1) 6 weeks intervention on a motor-driven treadmill REF [129]	1) Improvement of physical performance
**Quercetin, Resveratrol and Curcumin in Aging**			
**Antioxidant**	**Antioxidant Supplementation**	**Physical Activity Intervention**	**Outcome**
Quercetin	- 30 days 10, 25 and 50 mg/kg quercetin	1) Experiments performed on the evaluation of quercetin on cognitive performance REF [143]	1) Decreased oxidative stress and improvement of cognitive capacity
Resveratrol	- 13 weeks 0.2% (w/w) resveratrol	1) 5 days running program on a treadmill REF [145]	1) Improvement of aerobic exercise capacity
	- 10 days 0.05% resveratrol	2) 3 days of Isometric Exercise REF [146]	2) Reduction of the basal levels of oxidative stress
	- 12 weeks (500 mg/day) (w/w)	3) 12 weeks of aerobic training REF [151]	3) Increment of mitochondrial density, decreased muscle fatigue, increased resistance and reduced sarcopenia
Curcumin	- One month 0.2% (w/w) curcumin	1) One month wire monograph for detection of cerebral artery vasorelaxation REF [168]	1) Improvement of Aging-Related Cerebrovascular Dysfunction

In conclusion, these data suggest that an antioxidant supplementation with natural compounds, accompanied by a constant physical exercise session, represents a useful mean to reduce oxidative stress and to alleviate the age-related pathophysiological disorders. The exercise plan reinforces this concept by promoting physical well-being, by improving strength and physical performance in elderly subjects, potentially preventing the onset of sarcopenia and decreasing fatigue.

## CONCLUSIONS

ROS or free radicals derived from oxidative stress are required at low concentrations for many important physiological functions, such as muscle contraction and drug detoxification. However, the dramatic increase in ROS during strenuous physical exercise can damage cell membranes, having deleterious effects on skeletal muscle performance, macromolecule damage and cellular function impairment. A constant, progressive physical activity allows the cells to better detoxify a large amount of ROS, and this has been demonstrated both in adult subjects and in elderly people, who show antioxidant activity levels similar to young sedentary subjects and who can take advantage of regular physical activity to protect themselves from oxidative damage and prevent from age-related disorders. Besides the endogenous antioxidant systems, several natural compounds which are normally supplied within the diet can act as exogenous antioxidants and are marketed as important ergogenic factors in physical exercise, both in young age and in aging.

Therefore, exogenous natural supplements, together with a regular physical activity, are valid and promising molecules able to protect the body from oxidative damage and to alleviate the age-related pathophysiological disturbances. Future researches will further determine appropriate recommendations both in young age, adulthood and in aging especially, since nutrition and exercise are two effective and accessible strategies towards health maintenance in the aging population. Therefore, other natural, non-toxic compounds and innovations in research design may allow the opportunity to better understand the role of exogenous antioxidant supplementation and to give new, promising anticipations for the improvement of human healthcare.
